# Efficacy and safety of JAK inhibitors in the treatment of psoriasis and psoriatic arthritis: a systematic review and meta-analysis

**DOI:** 10.1186/s41927-022-00287-7

**Published:** 2022-09-27

**Authors:** Samantha Sarabia, Brandan Ranjith, Sahil Koppikar, Don Thiwanka Wijeratne

**Affiliations:** 1 School of Medicine, Queen’s University, 76 Stuart St, Kingston, ON Canada; 2 Faculty of Arts and Science, Queen’s University, 76 Stuart St, Kingston, ON Canada; 3 Division of Rheumatology, Department of Medicine, University of Toronto, 1 King’s College Circle, Toronto, ON Canada; 4 Division of General Internal Medicine, Department of Medicine, Queen’s University, 76 Stuart St, Kingston, ON K7L 2V7 Canada

**Keywords:** Psoriatic arthritis, Psoriasis, JAK inhibitors, Tofacitinib, Upadacitinib

## Abstract

**Background:**

JAK inhibitors are a relatively new class of medications that may be useful in the treatment of moderate-to-severe psoriasis and psoriatic arthritis (PsA). The objective of this study was to determine the efficacy of several JAK inhibitors in treating psoriasis and PsA and examine safety concerns.

**Methods:**

MEDLINE, Cochrane and EMBASE were searched for randomized controlled trials and observational studies comparing any JAK inhibitor to placebo. The primary outcomes were a 75% improvement in the Psoriasis Area and Severity Index (PASI75) and a 20% improvement in the American College of Rheumatology composite score (ACR20). A secondary outcome was the proportion of patients achieving a “0” or “1” on the static Physician Global Assessment scale. Odds ratios were used to compare the proportion of patients reaching these targets in the max dose intervention group vs. the placebo group. A random effects model was used to account for heterogeneity.

**Results:**

In total, 15 RCTs were included in the study and no observational studies. This encompassed 6757 patients in total. When the results were combined, the calculated odds ratio for PASI75 amongst tofacitinib vs. placebo was OR 14.35 [95%CI 7.65, 26.90], for PASI75 amongst non-tofacitinib JAK inhibitors vs. placebo it was OR 6.42 [95%CI 4.89, 8.43], for ACR20 amongst all JAK inhibitors versus placebo was OR 5.87 [95%CI 4.39, 7.85]. There was no significant difference in prevalence of serious adverse events between intervention and control in any of these studies.

**Conclusion:**

JAK inhibitors show promise for safely treating moderate-to-severe psoriasis and psoriatic arthritis.

**Supplementary Information:**

The online version contains supplementary material available at 10.1186/s41927-022-00287-7.

## Background

Psoriasis is an immune mediated skin disease affecting 1–3% of the general population [1]. It often manifests as erythematous, scaling plaques predominantly on the extensor surfaces of the body. Up to one third of patients with psoriasis develop psoriatic arthritis (PsA), which causes pain, stiffness and swelling of the joints and can lead to severe joint destruction and loss of function [2]. Patients with these conditions are at a significantly higher risk for metabolic syndrome, coronary artery disease, stroke, autoimmune diseases, depression, and many other conditions [3–5]. Psoriasis has been shown to have a great impact on quality of life in the majority of patients [6]. Skin and joint symptoms also affect fatigue levels and self-esteem. Early treatment is essential in both of these conditions to prevent morbidity and disability. Early treatment minimizes symptoms, prevents long term damage to the joints, reduces comorbidity burden, and improves quality of life [7]. NSAIDs and physiotherapy can be used to help manage arthritis symptoms, but they do not modify disease progression [8]. Topical ointments and phototherapy are common treatments for psoriasis, but do not usually control moderate to severe cutaneous disease. Patients with psoriasis and PsA often need treatment with conventional synthetic and biologic disease modifying anti-rheumatic drugs (DMARDs), however these medications have serious side effects and costs to consider. The same systemic DMARDs are often used to treat both psoriasis and PsA.

One therapy that has shown promise are the Janus Kinase Inhibitors (JAK), a type of small molecule targeted synthetic DMARD. Inhibition of the JAK/STAT pathway prevents the upregulation of pro-inflammatory genes involved in articular and extraarticular inflammation, by modulating cytokine signaling that are integral to lymphocyte activation, proliferation, and function. This class of medication provides an alternative therapeutic option for those that have an inadequate response to conventional DMARDs or biologic therapy [9, 10]. Furthermore, in rheumatoid arthritis, it has been shown to have relatively lower infection rates compared to biologic DMARDs [11–13].

JAK inhibitors have been studied in other conditions such as rheumatoid arthritis and ulcerative colitis [14, 15]. There have been systematic reviews on tofacitinib in psoriasis, however these reviews did not include other JAK inhibitors [16, 17]. Both of these reviews suggest that tofacitinib may be a treatment option for moderate-to-severe psoriasis and that the medication is generally well tolerated. A systematic review and meta-analysis has been completed on the efficacy and safety of various DMARDs for PsA, however it does not include non-tofacitinib JAK inhibitors [18]. The authors found that infliximab, guselkumab, adalimumab, golimumab, secukinumab and ustekinumab may be safer and more efficacious than the other targeted DMARDs evaluated in the study (including tofacitinib) for active PsA during induction therapy [8]. This systematic review and meta-analysis aims to widen the scope of review on tofacitinib, as well as provide an update and evaluate other JAK inhibitors, in both psoriasis and psoriatic arthritis. We evaluated randomized controlled trials and observational studies to outline the effectiveness and safety of JAK inhibitors in comparison to placebo so that they may be appropriately integrated into clinical practice, providing alternative therapy options for patients facilitating evidence-based practice and informed therapy selection. We also aimed to explore additional knowledge gaps such as differences in outcomes for older patients, those with immunocompromised status, as well as comparing different treatment timelines.

## Methods

### Eligibility criteria

We conducted a systematic review of primary research literature that included full-text, English language, original RCT’s and observational studies. Our population of interest was patients over 18 years who have been diagnosed with moderate to severe plaque psoriasis or PsA and are being treated with a JAK Inhibitor. We have excluded studies that evaluate topical JAK inhibitors based on intervention and we have excluded open label extension studies with no placebo arm based on study design. We determined that as placebo is the most homogenous comparator in clinical trials, it would be the most useful in allowing us to achieve our objective of determining the efficacy of JAK inhibitors. Hence studies were only eligible if the comparator was a placebo. See Additional file 2 Table S2 for components of the research question.

### Search strategy

**Fig. 1:**
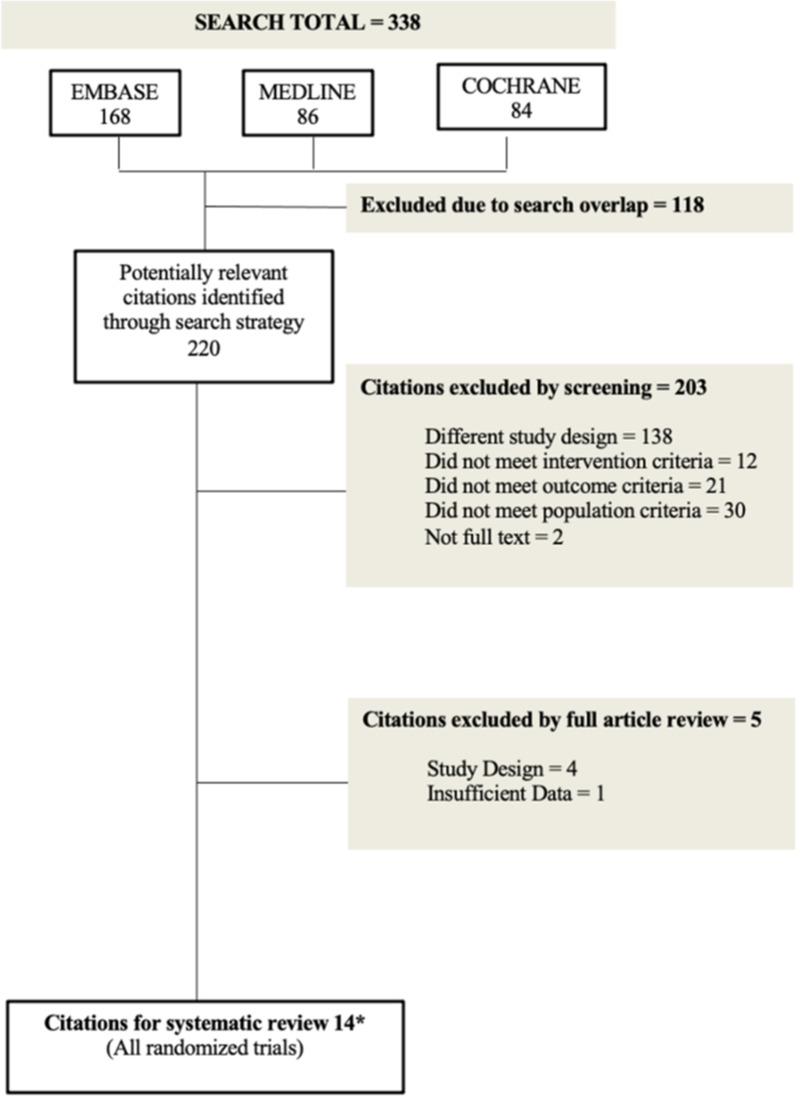
Literature Search Schema *Note 14 citations includes the SELECT PsA 1 and 2 trials, which were added to our analysis once the search was extended from January 12th 2019-May 4th 2021. OPT 1 and 2 trials were analyzed separately for a total of 15 trails

Electronic searches were performed on May 4th 2021 in MEDLINE, EMBASE and the Cochrane Register of Controlled Trials (CENTRAL) (Fig. 1). On March 1st 2022, the search was updated to include the period of May 5th 2021 to March 1st 2022. MeSH headings used were [“janus kinase inhibitor” “AND” “psoriasis”] “OR” [“janus kinase inhibitors” “AND” “psoriatic arthritis”]. All English Studies on humans from January 1999 to the March 2022 were included. No other filters or limits were used. All titles and abstract were reviewed by SS for inclusion based on the described criteria and verified independently by DTW. Full text screening was done by both these authors. The bibliography of included studies and the clinical trials.gov registry was screened for other potential eligible studies. Discrepancies were resolved by a third reviewer.

### Data extraction and outcome measures

Each included study was assessed in conjunction by two authors for data extraction and data extracted by each author was compared to the other. Any discrepancies were addressed by consensus between authors. The following outcomes were the only outcomes for which data was sought. The primary outcome was the proportion of patients who had a 75% improvement in their Psoriasis Area Severity Index Score (PASI75), a standard outcome measure for psoriasis [19]. A co-primary outcome was the American College of Rheumatology 20 (ACR20) score [20]. The secondary outcome was an improvement in static physician global assessment (sPGA) [19]. This standard outcome measure is a five point scale that measures the severity of psoriasis: 0 “clear”; 1 “almost clear”; 2 “mild”; 3 “moderate”, 4 “severe” [19]. The proportion of patients achieving a sPGA of 0 or 1 was considered a desirable outcome in our analysis. In regards to dactylitis and enthesitis, outcomes were measured by percent resolution as per the Dactylitis Severity Score (DSS) and percent resolution as per the Leeds Enthesitis Index (LEI) [21, 22]. Safety analysis evaluated the proportion of serious adverse events, herpes zoster infections, and venous thromboembolism in the treatment group with the maximum dose of the medication [23]. The outcome domains were selected because of their wide use across studies in psoriasis and PsA.

### Statistical analysis

For all outcomes, the counts of patients achieving each outcome and the number of patients in each treatment group was used as the numerator and denominator respectively to calculate proportions and percentages of patients achieving outcomes of interest for the interventional and control groups. Subgroup analysis was done on tofacitinib studies separately from non-tofacitinib JAK inhibitors as well as a separate analysis for phase III trials. Using these proportions, the odds ratio and 95% confidence intervals (CIs) were calculated for each comparison outcome. Subsequently, forest/funnel plots were created using the Cochrane Collaboration RevMan v5.3 software. When clinically appropriate and comparable study results were meta-analyzed. A random effects model was employed to account for heterogeneity of the meta-analyzed studies and p < 0.05 was considered to be statistically significant difference. Values of I^2^ > 50% and p < 0.10 were considered to indicate significant statistical heterogeneity. The included subgroup analyses were used in part to assess heterogeneity. When multiple doses were included in the studies analyses were performed on the maximum dose for each study.

### Bias assessment

Articles were independently assessed by each reviewer and dichotomized to low/high risk of bias based on standardized scoring systems (Table 2). An RCT was considered low risk if it satisfied a score of 8 or more based on the Cochrane Risk of Bias Tool Criteria [24].

## Results

Our search strategy yielded 338 total citations, of which 168 were from EMBASE, 86 from MEDLINE and 84 from COCHRANE. Of these citations, 118 duplicates were removed, leaving 220 for further evaluation (Fig. 1). Initial screening resulted in the exclusion of 203 studies, consisting of 138 that were excluded for study design, 12 did not meet intervention criteria, 21 did not meet outcome criteria, 30 did not meet population criteria and 2 were not full text. An additional 5 studies were excluded following full article review, 4 of which were excluded for study design and one for insufficient data. These studies captured 6757 patients. There were no studies that appeared to meet the inclusion criteria, but were excluded.

Of the included studies, 7 studies evaluated oral tofacitinib [10, 25–29] (Including the two part OPT study, which we have evaluated as two individual studies) and the remaining studies each evaluated INCB039110[30], GSK2586184[31], filgotinib [32], ASP015K [33], baricitinib [34], PF-04965842 [35] and upadacitinib [36, 37]. All studies compared the JAK inhibitor to placebos. In addition, Bachelez et al. compared tofacitinib to etanercept and Mease et al., 2017 also evaluated adalimumab, but these authors noted that they did not have sufficient power to determine superiority of etanercept or adalimumab vs. tofacitinib [28, 29]. [37] compared upadacitinib to adalimumab and found that both the 15 mg and 30 mg doses were noninferior to adalimumab and the 30 mg dose was superior[37]. The minimum treatment duration was 4 weeks and the maximum was 24 weeks. The follow up periods ranged from 8 to 52 weeks. Baseline characteristics can be found in Table 1. None of the studies reported significant differences in the proportion of participants in each group that was undergoing concomitant therapy. Additionally, some of the trials even stratified their groups based on this characteristic. Raw data can be found in Additional file 1: Table S1.

**Table 1:** Summary of included studies

Study ID and design	Study population characteristics (across all treatment and placebo groups)	Intervention	Control	Concommitent therapies permitted for trial	Duration of treatment and follow up of included outcomes	outcomes assessed	Serious adverse events in max dose and placebo groups	Herpes zoster cases on max dose
[28] Phase III RCT	Mean age: 44.0 Percent male: 71	N = 332 Tofacitinib (Max. dose 10 mg, BID)	N = 108 Placebo N = 336 Etanercept 50 mg 2x/wk (Data not included in analysis)	None. 2 week washout for topical and UVB treatment and at least 4 weeks for systemic therapies	12 weeks	PASI75 sPGA	5 (2%) on max dose; 2 (2%) on placebo	2
[30] Phase II RCT	Mean age: 48.4 Percent male: 64	N = 11 INCB039110 (Max. dose 600 mg, daily)	N = 12 Placebo	Stable dosing of topical therapy permitted. 4 week washout for unstable topical dosing, systemic therapies or phototherapy	4 weeks	PASI75 sPGA	None	None
[10] Phase III RCT	Mean age: 49.8 Percent male: 45	N = 132 Tofacitinib (Max. Dose 10 mg, BID)	N = 131 Placebo	Methotrexate, sulfasalazine, leflunamide permitted with max doses. 4 week washout period for TNFi	3 months before dose switch for another 3 months (only first 3 months data included), follow up at 2 weeks, 1 month, then monthly	PASI75 ACR20	3 (2%) on max dose; 3 (2%) on placebo	1
[31] Phase IIA RCT	Mean age: 44.0 Percent male 64	N = 14 ASP015K (Max. dose 400 mg, daily)	N = 14 Placebo	None. 2 week washout period for topical therapies, 4 weeks for phototherapy and 4–12 weeks for systemic therapies	4 weeks	PASI75 sPGA	1 (7%) on max dose considered to be treatment related	None
[37] Phase III RCT	Mean age: 50.8 Percent male: 48	N = 423 Upadacitinib (Max. dose 30 mg, daily)	N = 423 Placebo N = 429 Adalimumab 40 mg SC q2wks (Data not included in analysis)	≤ 2 Stable non-biologic DMARDs permitted with max doses. Patient may not be on both methotrexate and leflunamide. 4–12 week washout for TNFi, 2 weeks for topical therapy, 2–4 for phototherapy	24 weeks	PASI75 ACR20	26 (6.1%) on max dose; 13 (3.1%) on placebo	5
[29] Phase III RCT	Mean age: 47.7 Percent male: 44	N = 104 Tofacitinib (Max. dose 10 mg, BID)	N = 105 Placebo N = 106 Adalimumab 40 mg SC q2wks (Data not Included in analysis)	Methotrexate, sulfasalazine, leflunamide permitted with max doses. 6 month wash out for biologic DMARDs	3 months before dose switch for another 9 months (only first 3 months’ data included)	PASI75 ACR20	1 (1%) on max dose; 1 (1%) on placebo	None
[32] Phase II RCT	Mean age: 49.0 Percent male: 45	N = 65 Filgotinib (Max. dose 200 mg, daily)	N = 66 Placebo	Methotrexate, sulfasalazine, leflunamide and hydroxychloroquine permitted with max dose. 4–12 week washout for TNFi, 2 weeks for topical therapy, 4 for phototherapy	4 weeks	PASI75 ACR20	1 (2%) on max dose	1
[36] Phase III RCT	Mean age: 53.4 Percent male: 46	N = 218 Upadacitinib (Max. dose 30 mg, daily)	N = 212 Placebo	≤ 2 Stable non-biologic DMARDs permitted with max doses. Patient may not be on both methotrexate and leflunamide. 4–12 week washout for TNFi, 2 weeks for topical therapy, 2–4 for phototherapy	24 weeks	PASI75 ACR20	18 (8%) on max dose; 4 (2%) on placebo	8
[36] Phase IIB RCT	Mean age: 44.3 Percent male: 63.5	N = 49 Tofacitinib (Max. dose 15 mg, BID)	N = 50 Placebo	None. 4–12 week washout period for DMARDs, 2 weeks for topical therapy, 2–4 weeks for phototherapy	4 weeks	PASI75 sPGA	1 (1%) on max dose; 1 (1%) on placebo	None
[26] Phase IIA RCT	Mean age: 48.1 Percent male: 78.2	N = 17 ASP015K (Max. dose 100 mg, BID)	N = 29 Placebo	None. At least 8 week washout period for DMARDs, 1–2 weeks for topical therapies, 8 weeks for phototherapy	6 weeks	PASI75	None	None
[26] Phase III RCT OPT 1	Mean age: 45.8 Percent male: 70.8	N = 360 Tofacitinib (Max. dose 10 mg, BID)	N = 177 Placebo	None. 2–4 week washout for topical therapies or phototherapy, 4 weeks for etanercept and non-biologic DMARDs, 8–12 weeks for biologic DMARDs	16 weeks	PASI75 sPGA	10 (3%) on max dose; 5 (3%) on placebo	5
[33] Phase III RCT OPT 2	Mean age: 45.4 Percent male: 67.6	N = 381 Tofacitinib (Max. dose 10 mg, BID)	N = 196 Placebo	None. 2–4 week washout for topical therapies or phototherapy, 4 weeks for etanercept and non-biologic DMARDs, 8–12 weeks for biologic DMARDs	16 weeks	PASI75 sPGA	5 (1%) on max dose; 2 (1%) on placebo	1
[36] Phase IIB RCT	Mean age: 47.3 Percent male: 72.7	N = 69 (Max. dose 10 mg, daily) Baricitinib	N = 34 Placebo	None. 8 week washout period for biologic DMARDs, 4 weeks for non-biologic DMARDs or phototherapy, 2 weeks for topical therapies	12 weeks before dose switch for another 12 weeks (only first 12 weeks’ data included)	PASI75	1 (1%) on max dose; 1 (3%) on placebo	None
[35] Phase II RCT	Mean age: 45.6 Percent male: 68.0	N = 16 Tofacitinib (Max. dose 400 mg daily)	N = 14 Placebo	None. 4 week washout period for prohibited medications (undefined in manuscript)	4 weeks	PASI75 sPGA	0 (0%) on max dose; 1 (3%) on placebo	None
[27] Phase III RCT	Mean age: 41.1 Percent male: 72.9	N = 90, Tofacitinib (Max. dose 10 mg, BID)	N = 88 Placebo	None. Washout periods not included in manuscript	16 weeks before dose switch for another 36 weeks (only first 16 weeks’ data included)	PASI75 sPGA	None	3

## Primary outcome—psoriasis

**Fig. 2:**
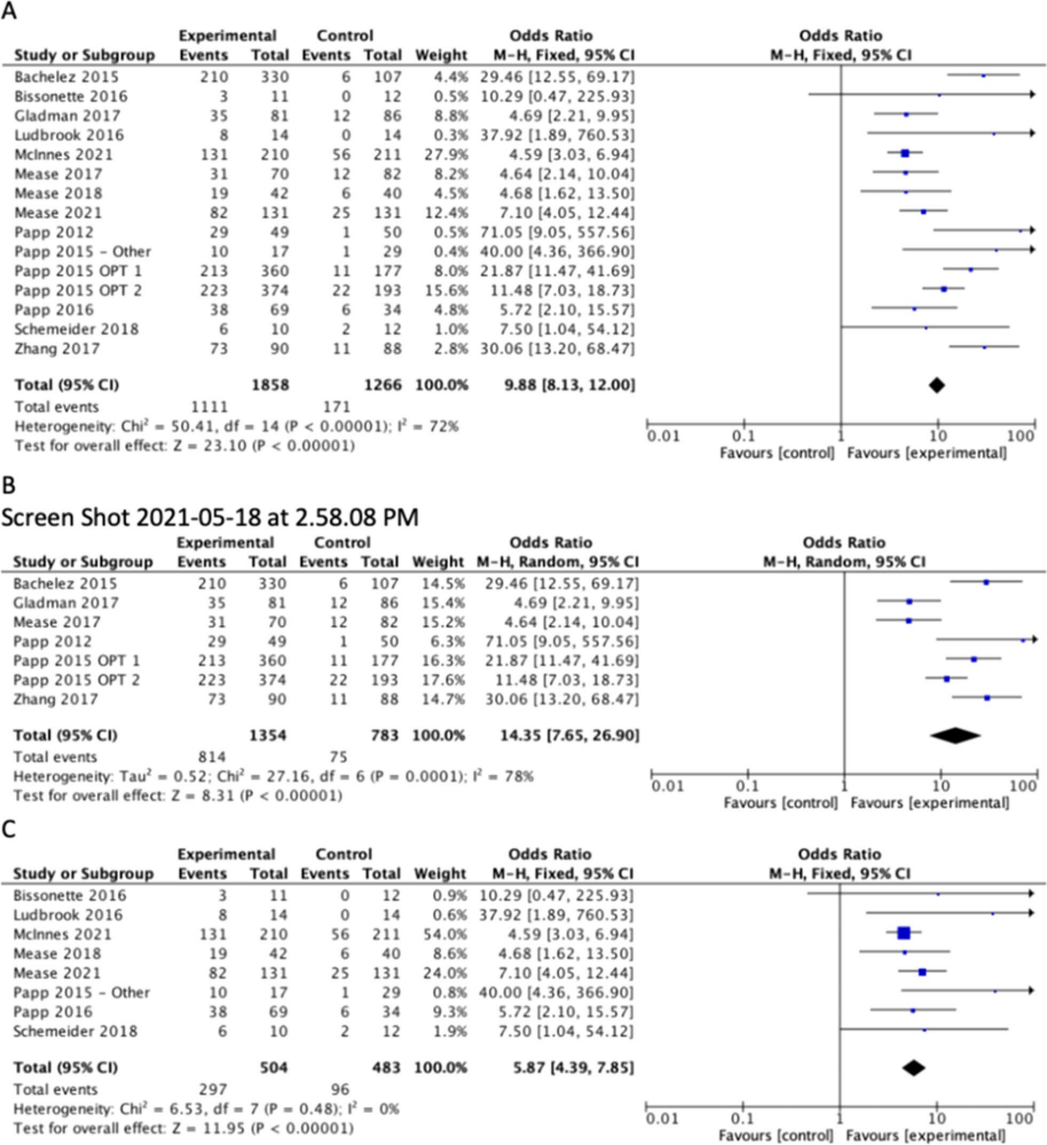
Difference in the proportion of patients achieving a 75% importance in the Psoriasis area and severity index (PASI75) between treatment and placebo for (**A**) All includes studies (**B**) Studies with tofacitinib as experimental group and (**C**) Studies with non- tofacitinib JAK inhitors as experimental group

All studies included PASI75 as an outcome. All of the studies that evaluated tofacitinib showed a significant improvement in the proportion of patients that reached PASI75. When the results were combined, the calculated odds ratio for PASI75 amongst all JAK inhibitors vs placebo was OR 9.88 [95% CI 8.13, 12.00] (Fig. 2a). Tofacitinib vs placebo was OR 14.35 [95% CI 7.65, 26.10] (Fig. 2b), showing significant improvement in PASI75 in the tofacitinib vs. placebo groups. The statistically heterogeneity was I^2^ = 78%, p < 0.001.

When comparing all other JAK inhibitors to placebo, Bissonette et al. was the only study that did not show a significant improvement in the proportion of patients achieving PASI75 [OR 10.29; 95%CI 0.47, 225.93]. When the results were combined, the calculated odds ratio for PASI75 amongst all non-tofacitinib JAK inhibitor studies vs placebo was OR 5.87 [95%CI 4.39, 7.85], showing significant improvement in PASI75 in the non-tofacitinib JAK inhibitors vs. placebo groups (Fig. 2c). The statistical heterogeneity was I^2^ = 0%, p = 0.48.

**Fig. 3:**
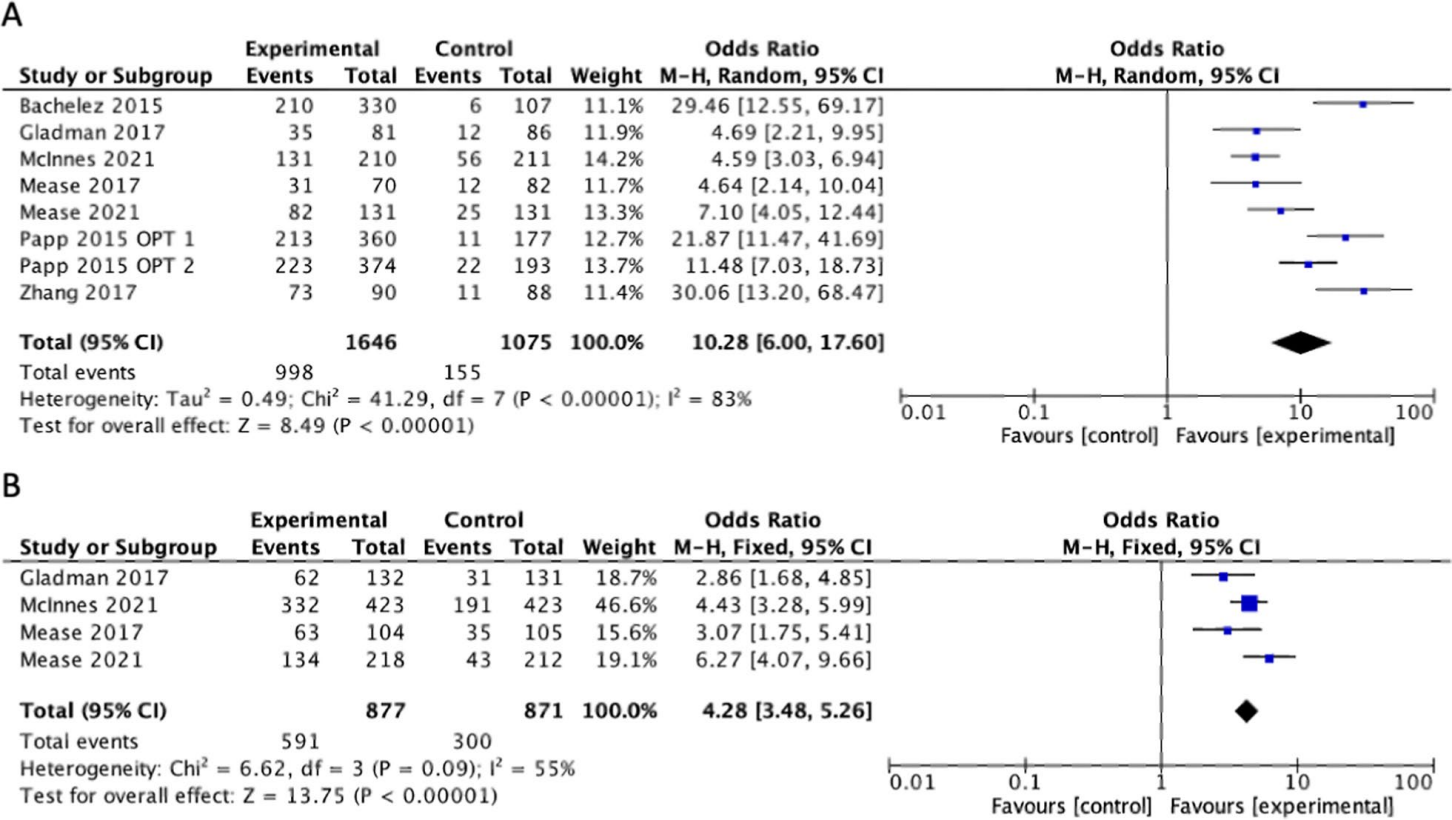
Subgroup analysis of phase III trials comparing difference in the proportion of patients achieving a (**A**) 75% improvement in the Psoriasis area and severity index (PASI75) between treatment and placebo and (**B**) 20% improvement in the American college of Rheumatology composite (ACR20) score between treatment and placebo

Additional subgroup analysis was completed on only the phase III RCTs for PASI75. When the results were combined, the calculated odds ratio amongst all JAK inhibitors vs placebo was OR 10.28 [95%CI 6.00, 17.60] (Fig. 3a). The statistical heterogeneity was significant (I^2^ = 83%, p < 0.001).

In regards to longer term follow up, Gladman et al. reported changes in PASI75 at 6 months were similar compared to the reported data at 3 months. Zhang et al. reported sustained PASI75 improvements at week 52 compared to week 16[10].

## Primary outcome—psoriatic arthritis

**Fig. 4:**
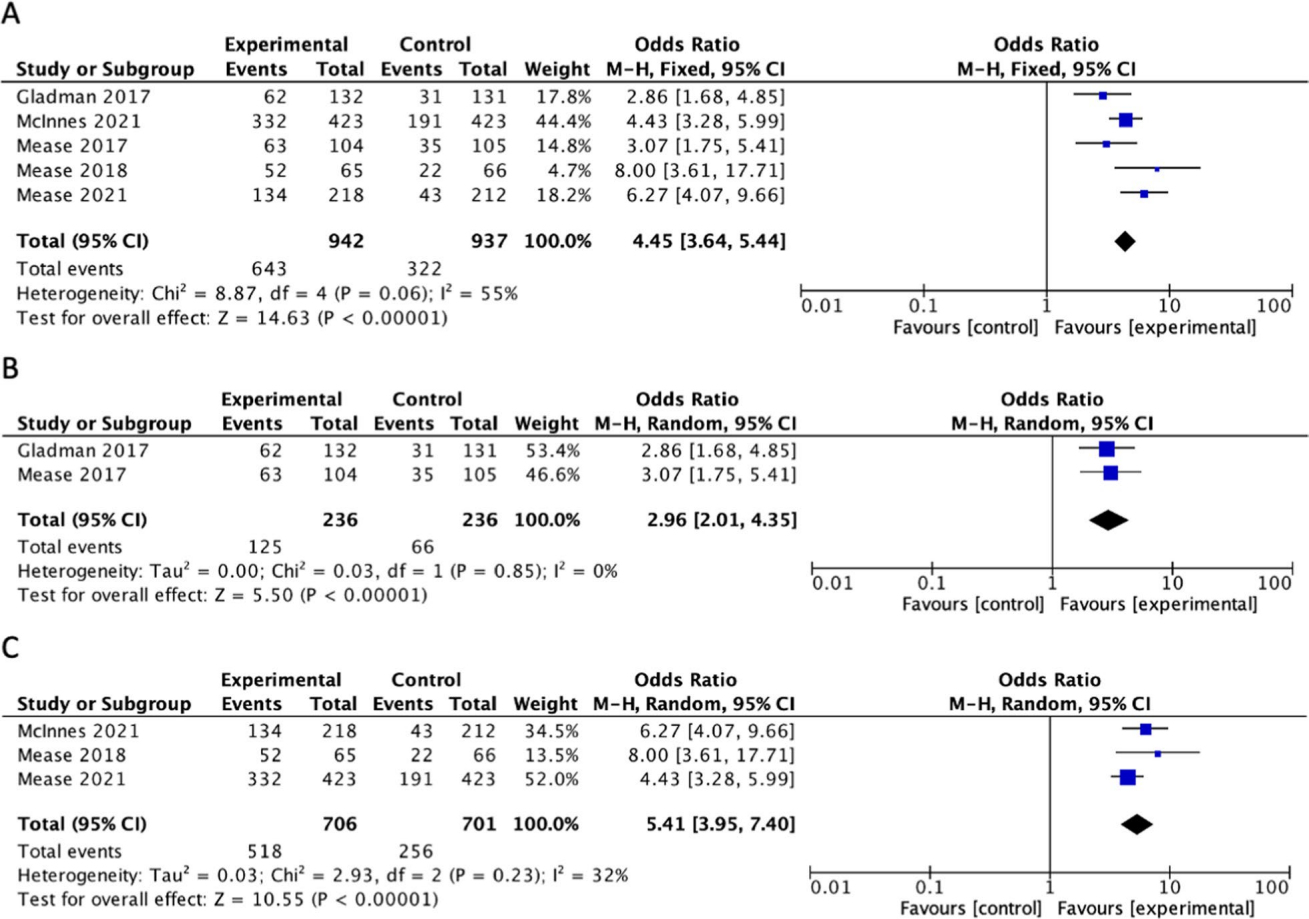
Difference in the proportion of patients achieving a 20% improvement in the American college of Rheumatology composite (ACR20) score between treatment and placebo for (**A**) All included studies (**B**) studies with tofacitinib as experimental group and (**C**) studies with non-tofacitinib JAK inhabitors as experimental group

Five of the studies included ACR20 as an outcome in patients with PsA. [32] evaluated response to filgotinib, McInnes et al. and [21] evaluated response to upadacitinib while Gladman et al. and [29] evaluated the response to tofacitinib[10, 29, 32, 36, 37]. Each of these studies showed a significantly higher proportion of patients achieving ACR20 in the treatment group compared to the placebo group. When the results were combined, the calculated odds ratio for ACR20 amongst all six studies vs placebo was 4.45 [95%CI 3.64, 5.44], showing significant difference between the proportion of patients that reached ACR20 in the treatment vs. placebo groups (Fig. 4a). The statistical heterogeneity was significant (I^2^ = 55%, p = 0.06). Subgroup analyses for tofacitinib and non-tofacitinib JAK inhibitors are displayed in Fig. 4b, c.

Additional subgroup analysis was completed on only the phase III RCTs for ACR20. When the results were combined, the calculated odds ratio amongst all JAK inhibitors vs placebo was OR 4.28 [95%CI 3.48, 5.26] (Fig. 3b). The statistical heterogeneity was significant (I^2^ = 55%, p = 0.09).

In regards to longer term follow up, Gladman et al. reported changes in ACR20 at 6 months were similar compared to the reported data at 3 months [10].

## Secondary outcomes

The sPGA response was measured in six of the studies (OPT 1 and 2 evaluated as separate studies) that evaluated response to tofacitinib, all of which had a significant difference between treatment and control groups [25–28]. When the results were combined, the calculated odds ratio for sPGA amongst all six studies for tofacitinib vs. placebo was OR 14.37 [95% CI 10.80, 19.10], showing significant difference between the proportion of patients that had an sPGA response in the treatment vs. placebo groups. The statistical heterogeneity was I^2^ = 0%, p = 0.48.

The sPGA response was measured in three of the studies that evaluated response to non-tofacitinib JAK Inhibitors. Bissonette et al. evaluated INCB039110, Ludbrook et al. evaluated GSK2586184 and Schemeider et al. evaluated PF-04965842 [30, 31, 35]. Each of these studies showed a significant difference in sPGA response in the treatment group compared to placebo. When the results were combined, the combined odds ratio amongst these three studies for non-tofacitinib JAK inhibitors vs. placebo was OR 16.67 [95% CI 3.82, 72.76], showing significant difference between the proportion of patients that had an sPGA response in the treatment and control groups. The statistical heterogeneity wasI^2^ = 0%, p = 0.92.

Outcome data on Enthesitis and dactylitis were only available in few studies. Gladman et al. did not have the power to test for statistical significance, but the results trend favorably in the same direction as their primary endpoints [10]. [32] did not analyze their dactylitis data because it was not uniformly scored at all centers [32]. In regards to enthesitis, they found enthesitis resolution at 16 weeks occurred in 26% more of the filgotinib treatment patients vs placebo [95% CI 4.0, 45.1], (p = 0.0089) [32]. McInnes et al. could not analyze dactylitis treatment response rate of upadacitinib vs placebo due to failure of hierarchy [37]. In regards to enthesitis, the upadacitinib treatment response difference vs placebo was 25.3%[95% CI 16.9,33.7], p < 0.001 [37]. Mease et al., 2021 reported their dactylitis response rate difference at 12 weeks between upadacitinib treatment and placebo as 40.1% [95% CI 23.4,56.7], (p < 0.001) and enthesitis was 27.9 [95% CI 17.6,38.2], (p < 0.001) [36].

Most of the studies included in this review had serious adverse events at a very low frequency (1–7% in the max dose intervention group). These serious adverse events were commonly infections and details can be found in Table 1. None of studies reported significantly more serious adverse events vs. the placebo group. Non-serious adverse events were most commonly nasopharyngitis or upper respiratory tract infections. Of special interest is the prevalence of herpes zoster virus in patients treated with JAK Inhibitors; details of which may also be found in Table 1. Eight of the studies reported herpes zoster infections in the max dose intervention group. There was limited data on rate of venous thromboembolism (VTE). [32] reported no cases with filgotinib; McInnes et al. reported one case with 30 mg upadacitinib, one with adalimumab and one with placebo; and Mease et al. 2021 reported one case in the 15 mg upadacitinib group [32, 36, 37].

The safety data reported in Table 1 reflects the adverse effects that occurred during the treatment duration. During longer term follow up, Gladman et al. reports that frequency of serious adverse events from baseline to 6 months were similar to those at 3 months [10].[34] did not find any increase in serious adverse events when comparing those participants who were changed from low dose to high dose barcitinib for weeks 12–24 versus those staying on the same dose [34]. Zhang et al. reported data from week 52 after a tofacitinib dose switch at week 15 and found that four patients receiving tofacitinib 5 mg BID, two receiving tofacitinib 10 mg BID, and one patient who advanced to tofacitinib 10 mg BID from placebo had serious AEs [27]. [29] also had a tofacitinib dose switch at 3 months for another 9 months. At the 12 month mark the placebo to 10 mg group had 4 (7.5%) participants with serious adverse events and none with herpes zoster [29]. The tofacitinib group treated entirely with 10 mg had 4(3.8%) participants with serious adverse events and 1(0.9%) with herpes zoster [29].

There was inadequate data to explore subgroups of interest aside from those previously noted. None of the studies stratified data by age or by other immunocompromised states, including those with diabetes. The maximum duration of placebo-controlled treatment was 24 weeks and the maximum duration of follow up was 52 weeks. More data is needed to compare short term and long-term outcomes.

The outcome of the Cochrane bias assessment can be found in Table 2. All studies were deemed to be low risk of bias. The funnel plots were symmetric, indicating minimum publication bias. No observational studies were captured from our searches. The symmetrical nature of the funnel plot is another indicator of the low risk of bias (Additional file 3. Fig. S1, Additional file 4 Fig. S2).

**Table 2:** Cochrane Risk of Bias Table for RCTs

Cochrane risk of bias tool criteria								
	Selection bias	Performance bias	Detection bias	Attrition bias	Reporting bias	Other bias	Total	
Study	*Random Sequence Generation*	*Allocation Concealment*	*Blinding of Participants & Personnel*	*Blinding of Outcome Assessment*	*Incomplete Outcome Data*	*Selective Reporting*	*Other Sources of Bias*	
[28]	2	2	2	2	2	2	2	14
[30]	1	1	2	2	2	2	2	12
[10]	2	2	2	2	2	2	2	14
[31]	2	2	2	2	2	2	2	14
[37]	2	2	2	2	2	2	2	14
[29]	2	2	2	2	2	2	2	14
[32]	2	2	2	2	2	2	2	14
[36]	2	2	2	2	2	2	2	14
[26]	2	2	2	2	2	2	2	14
[33]	1	1	2	2	2	2	2	12
[49]	2	2	2	2	2	2	2	14
[34]	2	2	2	2	0	2	2	12
[35]	2	2	2	2	2	2	2	14
[27]	2	2	2	2	2	2	2	14

0 = high risk

1 = unclear

2 = low risk

## Discussion

This review caputes a wider range of studies than previously published reviews, including the studies that focused on non-tofacitinib JAK Inhibitors. We have also included the most up to date clinical trial evidence that has not previously been included in systematic reviews and meta-analyses published to date. Our systematic review and meta-analysis investigates the efficacy and safety of JAK inhibitors in the treatment of moderate to severe plaque psoriasis and psoriatic arthritis. This study reveals that both tofacitinib and non-tofacitinib JAK Inhibitors are effective in treating psoriasis as measured by the PASI75 and sPGA, as well as PsA as measured by the ACR20, when compared to placebo. In addition, Bachelez et al. found tofacitinib to be non-inferior to the comparator etanercept ([29] did not have sufficient power to compare tofacitinib and adalimumab) in plaque psoriasis. Furthermore, McInnes et al. recently demonstrated in their clinical trial that upadacitinib was non-inferior to active comparator adalimumab in PsA for the ACR20 response. Interestingly, there seemed to be an overall trend towards better effect of non-tofacitinib JAKi compared to tofacitinib in PsA. However, it is important to note that the effect sizes cross over, and the individual studies did not compare tofacitinib to other JAKi. Therefore, direct comparisons cannot be made, and we cannot definitively conclude that non-tofacitinib JAKi are superior. Regardless, these findings are promising for those patients who need an alternative to currently existing biologic DMARD therapy.

Our results are consistent with the findings by [16] systematic review and meta-analysis looking at tofacitinib and its efficacy and safety in treating moderate to severe psoriasis as measured by PASI75 and sPGA. However they had a total population of 2724, which is substantially smaller than the 6757 patient included in the studies analyzed here [16]. A systematic review by [17] also reported similar results, with a combined adjusted risk ratio for PASI75 amongst tofacitinib 10 mg vs placebo of RR 7.30 [95% CI 5.55, 9.59], whereas our odds ratio for max dose tofacitinib vs placebo was OR 14.35 [95% CI 7.65, 26.90] and Kuo reported a risk difference of RD 0.51 (95% CI 0.43–0.58) [16, 17]. One of the studies included in the present systematic review also found that tofacitinib 10 mg twice daily was superior to placebo and non-inferior to subcutaneous etanercept 50 mg twice weekly [28] in treating psoriasis. An ad hoc analysis done by Mamolo et al. of the phase IIb tofacitinib trial done by Papp et al., outlined the patient reported outcome measure results of the study. They reported a significant improvement in psoriasis as measured by the dermatology quality of life index (p < 0.05), the SF-36 mental component score (p < 0.05) and proportion of PGA scores at 0 or 1 (p < 0.0001) at 12 weeks for all drug doses compared to placebo [38]. It has also been shown that tofacitinib provides relief from pruritis associated with psoriasis [38].

From a safety perspective, tofacitinib is the most well studied JAK inhibitor currently, and there has been concern about the possible higher prevalence of herpes zoster virus infections and the need for prophylactic vaccination and clinical monitoring in patients taking this drug [39, 40]. Unfortunately, many of these studies provided insufficient data to evaluate the risk of VTE in treatment with JAK inhibitors. [32] reported no cases with filgotinib; McInnes et al. reported one case with 30 mg upadacitinib, one with adalimumab and one with placebo; and [36] reported one case in the 15 mg upadacitinib group [32, 36, 37]. Furthermore, the study by Kuo et al. noted that the rate of some other adverse events was higher in the 10 mg BID tofacitinib group than the placebo group, including upper respiratory tract infections, hypercholesterolemia, elevation in creatinine phosphokinase (CPK), and headache. [16]. While our safety analysis focused on serious adverse events and herpes zoster infections, the studies included in this systematic review have similar rates of the adverse events noted in the Kuo et al. review. A previous systematic review for tofacitinib in rheumatoid arthritis (RA) showed that studies reported a rate of serious adverse events in the range of 0–5.9%, which is comparable to the range shown by the studies in this systematic review of 0–7% for all JAK inhibitors (0–3% for tofacitinib only) [41]. This suggests that JAK inhibitors have a similar safety profile in both PsA and RA. In most jurisdictions, only the lower doses of the JAK inhibitors, which have a better safety profile, have been approved for PsA. However, long term safety data will still be needed in this class, especially in light of early results from the ORAL Surveillance study in RA which suggested higher rates of major cardiovascular events and malignancies in patients taking tofacitinib compared to TNF inhibitors [42].

Despite the efficacy of JAK inhibitors, this safety data will be important for clinicians and patients to consider while deciding upon appropriate advanced targeted therapies, especially given a choice of other therapeutic classes and their long-term safety data, such as TNF inhibitors.

The use of tofacitinib in PsA has not been as extensively studied as in psoriasis. A recent systematic review and meta-analysis compared a number of DMARDs and found that tofacitinib improved ACR20, as shown in our study [18]. The combined calculated odds ratio for ACR20 amongst studies comparing tofacitinib vs. placebo was OR 2.75[95% CI 1.96, 3.86], whereas our study determined it to be OR 4.45 [95%CI 3.64, 5.44] for all JAK inhibitors for which this was measured, noting a possible improvement [18]. The same study also evaluated PASI75 and found the combined odds ratio to be OR 3.63[95% CI 2.19, 6.02], whereas our study determined it to be OR 14.35[95% CI 7.26, 26.90] for tofacitinib, noting a significant improvement [18]. These differences may be explained by the fact that Lu et al. only looked at tofacitinib and not filgotinib or upadacitinib and therefore had fewer studies than our analysis. The higher OR in our study appears to be primarily driven by filgotinib. The open label extension study OPAL BALANCE, which evaluated eligible patients from the phase III OPAL BROADEN and OPAL BEYOND studies, was not included in this analysis, however the 36 month interim analysis concluded that the safety profile of tofacitinib was similar to that reported in OPAL BEYOND (TNF inhibitor naïve patients) and OPAL BROADEN (patients with an inadequate response to TNF inhibitors), which were included in this systematic review [10, 29, 43].

Overall, patient reported outcome data from the OPAL BEYOND trial also showed improvements exceeding placebo in several patient reported, functional and quality of life measures [44]. The results were similar in the OPAL BROADEN trial as well as the SELECT PsA 1 and 2 trials for upadacitinib [36, 37, 45]. These studies also evaluated enthesitis and dactylitis. OPAL BROADEN could not declare statistical significance for these measures due to the hierarchical testing scheme used, however the observed effects of tofacitinib were in the same direction as the primary endpoints. OPAL BEYOND showed a significantly greater decrease in the Leeds Enthesitis Index score in the 10 mg tofacitinib group vs placebo (p < 0.001). Hierarchical statistical testing failed for the 5 mg group, so according to the hierarchy, dactylitis could not be tested, however the observed effects of tofacitinib were in the same direction as the primary endpoints. The 36-month interim report for the OPAL balance study reported that changes in the Leeds Enthesitis Index and Dactylitis Severity Score were maintained up to month 30. Statistics for these measures were not reported. Upadacitinib also showed superiority compared to placebo in enthesitis resolution in both SELECT PsA studies at 24 weeks. It was also superior to placebo for dactylitis resolution in the SELECT PsA-2 study (biologic non-responders),but could not be analyzed in SELECT PsA-1 due to failure of hierarchical analysis they had in the study [36, 37].

The SELECT long term extension study showed that present achieving PASI75, ACR20, complete resolution of enthesitis and complete resolution of dactylitis remained consistent or improved at week 56 [46]. Taken together, these studies suggest that both tofacitinib and upadacitinib work in treating most musculoskeletal endpoints in PsA. While long term evidence is somewhat limited, there are trials showing lasting benefits. Furthermore, the significant improvements in patient related and functional outcomes seen in the studies is reassuring, as the aim is to improve quality of life in addition to preventing damage in PsA. Given that PsA is such a clinically heterogenous disease, which can be challenging to treat as therapies that improve skin may not improve musculoskeletal manifestations, having JAK inhibitors show effectiveness in multiple domains of PsA is promising and provides another option for clinicians.

Most studies evaluating non-tofacitinib JAK inhibitors are almost all phase II trials, as these medications are in earlier stages of development than tofacitinib, except for upadacitinib which recently completed phase III trials and provides a potential alternative. As seen in Fig. 3, the PASI75 data is not as unified as tofacitinib, with the Bissonette et al. study on INCB039110 not showing statistical significance, however most of the other medications show promise. There are no other systematic reviews looking at the efficacy or safety of these medications and further study is needed to determine the role they may play in the treatment of psoriasis and PsA once more phase III trials are published.

We are limited by the lack of obervational studies, and thus real world data. Curretly data is limited to maximum of 24 weeks of placebo control treatment and 52 weeks of follow-up in most cases, limiting information on medium to longterm safety and efficany outcomes. However this review includes 15 high quality RCTs with over 6000 patients from phase 2 and 3 clinical trials. We did also encounter high statistical heterogeniety in some of our anaylses, which we believe can be attributed to the large range of sample sizes across the studies. No significant clinical hetergeneity could be identified. However it is possible that there may be differences in patient population based on DMARD failures and biologic failures making them eligible to take the study medications. For example, the biologic failure studies tend to have slightly higher disease severity as expected, although this information is not clearly extractable from the studies.

This review has shown that JAK inhibitors are a promising class of medications for the treatment of moderate to severe plaque psoriasis and PsA through the evaluation of PASI75, ACR20 and sPGA response. Our results align with previous litrature. In this review we qauntify this improvement as a 14.4 fold improvement in psoriatic plaques over plaebo, as measured by PASI75 for tofacitinib and 4.5 fold improvement in PsA over placebo as measured by ACR20 for both included JAK inhibitors. This is interesting, considering the fact that no JAK inhibitors have been approved for use in psoriasis. This could potentially be explained by the fact that the standard efficacy target for new biological treatments for psoriasis is PASI90 or PASI100. While direct comparisons cannot be made between studies, current data does not indicate that JAK inhibitors will outperform other biologic classes (i.e. IL-17, IL-12/23 or IL-23 inhibitors) in skin outcomes [47, 48]. However, the efficacies in skin outcomes seen in some JAK inhibitor studies, such as the recent SELECT PsA-1 trial, are better than those observed for other oral treatments such as apremilast and some older biologics such as etanercept, suggesting it may have a role in certain patients [49–51].

Overall, JAK inhibitors provide a novel and different mechanism of action compared to previous therapies for psoriatic disease, having a combined effect on multiple cytokines through their action on the JAK enzymes. The phase III trials show that both tofacitinib and upadacitinib can be potentially used as the first targeted therapy or after TNF-inhibitor failure [36, 37, 44, 45]. Unfortunately, as stated above, both tofacitinib and upadacitinib seem to not be as effective as the IL-17, IL-12/23, or IL-23 agents against moderate-to-severe skin disease, although head-to-head trials have not been done. Based on these findings, for patients with PsA with significant joint disease, but milder skin disease, certainly a JAK inhibitor could be the drug of choice. Therefore, JAK inhibitors could conceivably have a place in the treatment algorithm for psoriatic disease because they are oral treatments or if other considerations, such as arthritis, dactylitis or enthesitis are present in the context of milder psoriasis.

## Conclusion

Our study supports the use of JAK inhibitors as an alterative therapy for those who have not been succesfuly treated with other biologic DMARDS or for those who prefer oral to injectable medications for both skin and joint disease. More research will need to be done to directly compare JAK inhibitors to each other and to other therapies with different mechanisms of action to determine their optimal role in treating psoriatic disease and its various manifestations. Data will be needed on whether JAK inhibitors can be used as monotherapy or whether they need background conventional DMARDs to be effective. The post marketing information on these medications is limited and more data will be needed to ensure the safety and efficacy of JAK inhibitors in the long term. Further research will also be required on other patient subgroups, including older patients and those with comorbid immonocompromising conditions such as diabetes and chronic kidney disease. This information will be important to estimate real word effects and impact of these therapies.

## Supplementary Information

**Additional file1**: **Table S1.** Raw data for outcomes.

**Additional file2**: **Table S2.** Summary of research question

**Additional file3**: **Fig. S1.** Funnel plot for studies that include 75% improvement in the Psoriasis area and severilty index (PASI75) as an outcome

**Additional file4**: **Fig.S2** Funnel plot for studies that included 20% improvement in the American college of Rheumatology composite score (ACR20) as an outcome

## Data Availability

The datasets used and/or analyzed during the current study are available from the corresponding author on reasonable request.

## Abbreviations

PsA: Psoriatic arthritis
NSAIDs: Non-steroidal, anti-inflammatory drugs
DMARD: Disease modifying anti-rheumatic drugs
JAK: Janus kinase
JAKi: Janus kinase inhibitors
STAT: Signal transducer and activator of transcription
RCT: Randomized control trial
CENTRAL: Cochrane register of controlled trials
PAS: Psoriasis area severity index score
ACR20: American college of rheumatology
LEI: Leeds enthesitis Index
CI: Confidence intervals
OR: Odds ratio
sPGA: Static physician global assessment
RD: Risk Difference
RR: Relative risk
SF-36: 36-Item short form survey
VTE: Venous thromboembolism
CPK: Creatinine phosphokinase
RA: Rheumatoid arthritis
TNF: Tumor necrosis factor
