# Supplementary figures and images for: Efficacy and safety of JAK inhibitors in the treatment of psoriasis and psoriatic arthritis: a systematic review and meta-analysis
Source: BMC Rheumatol. 2022 Sep 27;6:71. doi: 10.1186/s41927-022-00287-7 (PMC9513929; doi:10.1186/s41927-022-00287-7)

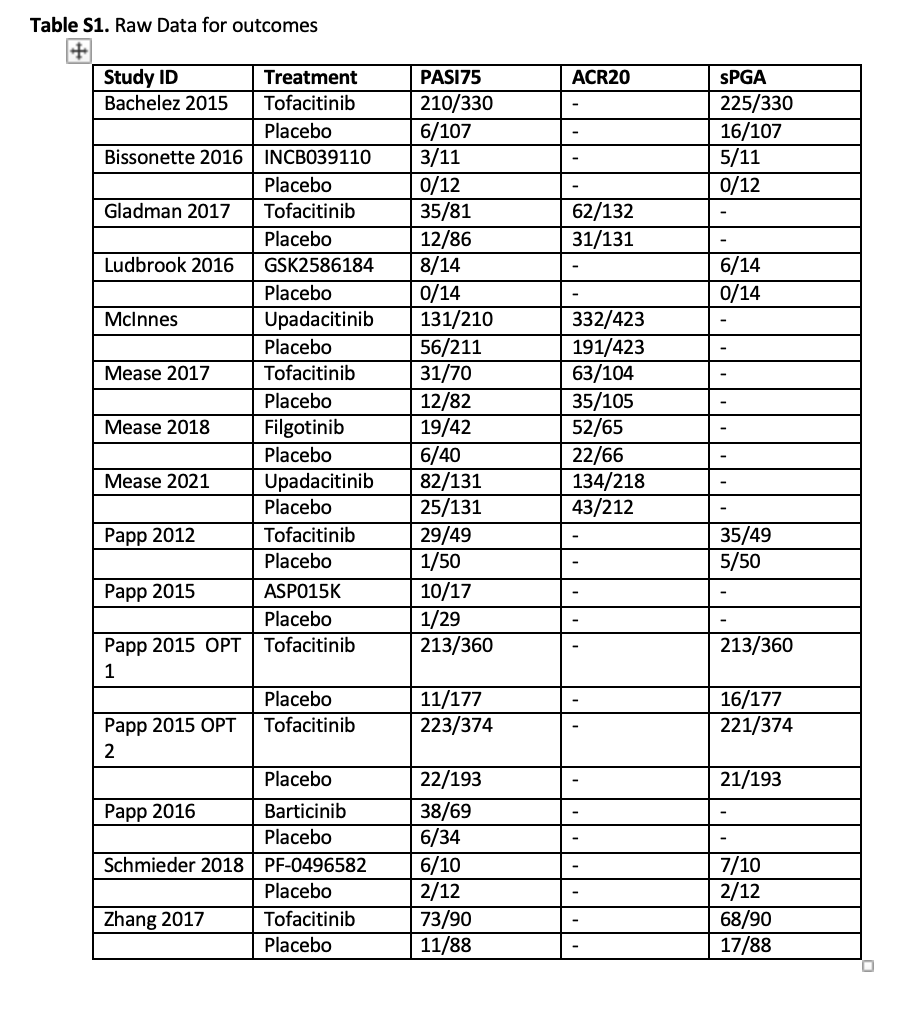

Supplement: Supplementary file 1 — Additional file1: Table S1. Raw data for outcomes. [file 41927_2022_287_MOESM1_ESM.docx]

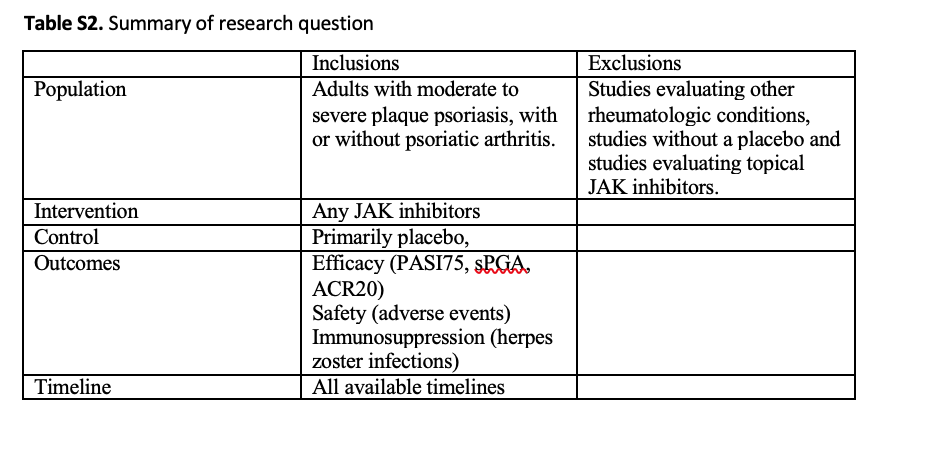

Supplement: Supplementary file 2 — Additional file2: Table S2. Summary of research question [file 41927_2022_287_MOESM2_ESM.docx]

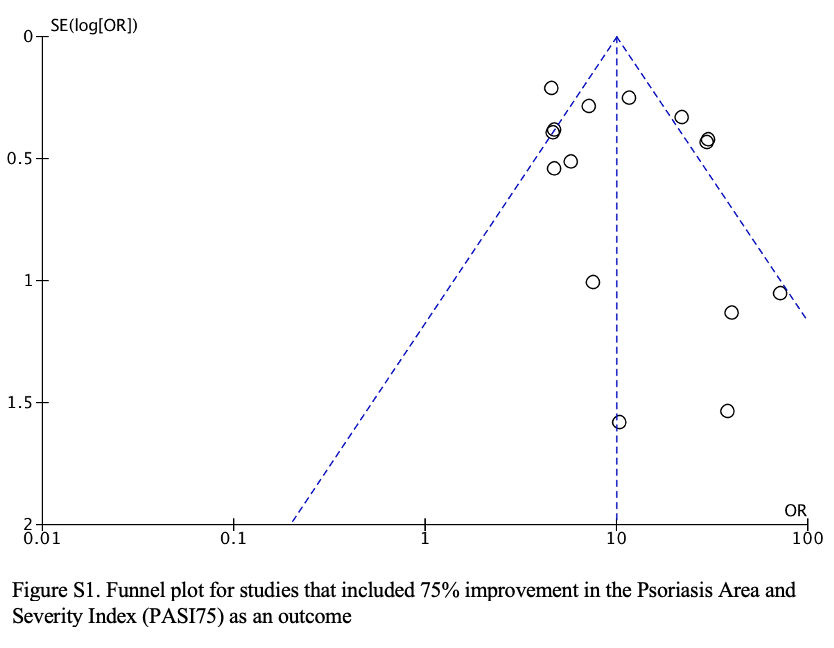

Supplement: Supplementary file 3 — Additional file3: Fig. S1. Funnel plot for studies that include 75% improvement in the Psoriasis area and severilty index (PASI75) as an outcome [file 41927_2022_287_MOESM3_ESM.docx]

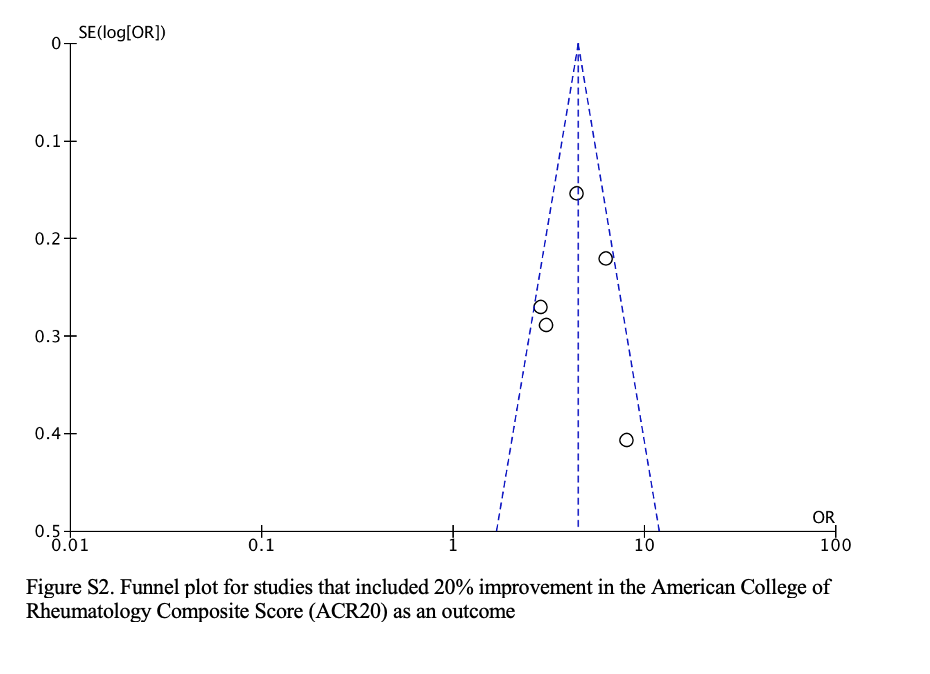

Supplement: Supplementary file 4 — Additional file4: Fig.S2 Funnel plot for studies that included 20% improvement in the American college of Rheumatology composite score (ACR20) as an outcome [file 41927_2022_287_MOESM4_ESM.docx]
